# Non-immersive virtual reality games, sleep, and sedentary behavior in children

**DOI:** 10.3389/fspor.2026.1775637

**Published:** 2026-04-07

**Authors:** Ana Patrícia da Silva Souza, Sandra Lopes de Souza, Maria Eduarda Rodrigues Alves dos Santos, Ana Beatriz Januário da Silva, Karollainy Gomes da Silva, Robson Feliciano da Silva, José Maurício Lucas da Silva, Mayara Luclécia da Silva, Érica Helena Alves da Silva, Williclecia Walkiria Dias Ferreira, Paulo Roberto Leite de Arruda, Antonietta Cláudia Barbosa da Fonseca Carneiro, Waleska Maria Almeida Barros

**Affiliations:** 1 Postgraduate Program in Neuropsychiatry and Behavioral Sciences, Center of Medical Sciences, Federal University of Pernambuco, Recife, Brazil; 2 Department of Physiotherapy, Center of Health Sciences, FACOL University Center (UNIFACOL), Vitória de Santo Antão, Brazil; 3 Integrated Center of Neuroscience Technologies, FACOL University Center (UNIFACOL), Vitória de Santo Antão, Brazil; 4 Department of Medicine, Center of Health Sciences, FACOL University Center (UNIFACOL), Vitória de Santo Antão, Brazil

**Keywords:** children, physical activity, sedentary behavior, sleep, virtual reality

## Abstract

**Introduction:**

Short sleep duration in children may lead to an energy imbalance by disrupting hormonal regulation, reducing physical activity levels, increasing sedentary behaviors, and elevating caloric intake. Physical activity has been proposed as an effective non-pharmacological strategy to improve sleep outcomes. Virtual reality can increase enjoyment during exercise, potentially boosting engagement in physical activity.

**Aim:**

To evaluate the effects of non-Immersive virtual reality gaming on sleep duration and sedentary behavior time in children.

**Methods:**

Prospective longitudinal study with children aged 5 to 9 years over eight weeks. Sleep duration was assessed using a four-item questionnaire, and sedentary behavior using the Cardiovascular and Environmental Questionnaire. A linear mixed-effects model was applied.

**Results:**

No significant effects were observed for group, time, or their interaction on sleep duration (*p* = 0.173; 0.380; 0.839) or sedentary behavior (*p* = 0.070; 0.088; 0.895). Sixty-two children (mean age 6.74 ± 1.51 years; 29 girls) from five schools were randomly assigned to a control (*n* = 28) or virtual reality group (*n* = 34). Age-adjusted mixed models showed mean sleep duration of 620 min (control) vs. 601 min (virtual reality) and sedentary behavior of 340 min vs. 390 min, respectively.

**Conclusion:**

There were no significant changes in sleep duration or in time spent sedentary. The absence of effects may be related to the intervention dose and the sensitivity of the self-report instruments, highlighting the need for further studies using larger samples, objective measures, and interventions that promote healthier movement behaviors in children.

## 1 Introduction

School-aged children are sleeping less than in previous decades. This reduction in sleep duration is likely associated with contemporary lifestyle habits, including excessive exposure to artificial light, increased screen time, caffeine consumption, and the absence of consistent bedtime routines (1). According to the National Sleep Foundation guidelines, children aged 3 to 5 years should obtain 10–13 h of sleep per night, whereas those aged 6 to 10 years are recommended to sleep 9–11 h per night (2).

Healthy sleep is essential for the physical and emotional well-being of children and encompasses adequate sleep duration and continuity, regular sleep onset and wake times, and minimal sleep disturbances (3). Concerns have been raised regarding this population due to the risk of developing persistent sleep patterns characterized by delayed sleep onset and reduced sleep duration (4). Short sleep duration contributes to an energy imbalance by disrupting hormonal regulation, reducing physical activity levels, increasing sedentary behavior, and elevating caloric intake (5–7). In this context, physical activity has been proposed as an effective non-pharmacological strategy to improve sleep quality (8).

Virtual reality (VR) integrates physical exercise with game-based activities and has increasingly been explored for clinical and behavioral outcomes in both adults and children (9, 10). VR games provide additional cognitive stimulation through their dual-task characteristics and may enhance enjoyment during exercise, thereby encouraging greater engagement in physical activity (11). Spending more time in bed or increasing total sleep duration does not necessarily ensure better sleep quality or efficiency; however, engaging in moderate to vigorous physical activity has been shown to enhance both sleep duration and efficiency (12). In this context, interventions incorporating gamification strategies may promote higher-intensity physical activity by increasing engagement and motivation during gameplay. Evidence suggests that such interventions can increase moderate-to-vigorous physical activity levels among children and adolescents, with effects that may persist beyond the intervention follow-up period (13). However, few studies have investigated the effects of VR interventions on sleep outcomes in children. Although a study involving children with chronic disease demonstrated improvements in sleep duration following a 12-week intervention (14), evidence regarding the effects of VR interventions on sleep in healthy pediatric populations remains limited. Furthermore, the potential of non-immersive VR game-based interventions implemented in school settings to influence sleep and sedentary behavior in children remains largely unexplored. Therefore, the present study aimed to examine the effects of a non-immersive VR game-based intervention on sleep duration and sedentary behavior in children.

## 2 Methods

### 2.1 Study design and participants

This study employed a prospective longitudinal interventional design. The sample comprised children of both sexes, aged 5 to 9 years, enrolled in municipal public schools. Data collection and the intervention were conducted in five schools randomly selected using the Research Randomizer program (version 3.0). Children with physical or mental disabilities or conditions that could limit their ability to perform the assessments were excluded. Additionally, children diagnosed with chronic diseases, including hypertension, diabetes mellitus, or heart disease, were not eligible for participation.

The study was approved by the Human Research Ethics Committee of the Federal University of Pernambuco (CAAE: 58651822.8.0000.5208; approval number: 5.586.951). Data collection took place between February 2023 and August 2025. Parents or legal guardians received detailed information about the study objectives, procedures, risks, and benefits and provided written informed consent. Children also provided assent through a separate form. Participants were allocated to the Control (C) or Virtual Reality (VR) groups using simple randomization conducted by an independent researcher. Group assignments were concealed in sequentially numbered, opaque, sealed envelopes prepared in advance. Each envelope was opened only after the participant had been enrolled in the study.

#### 2.1.1 Procedures

**Figure 1:**
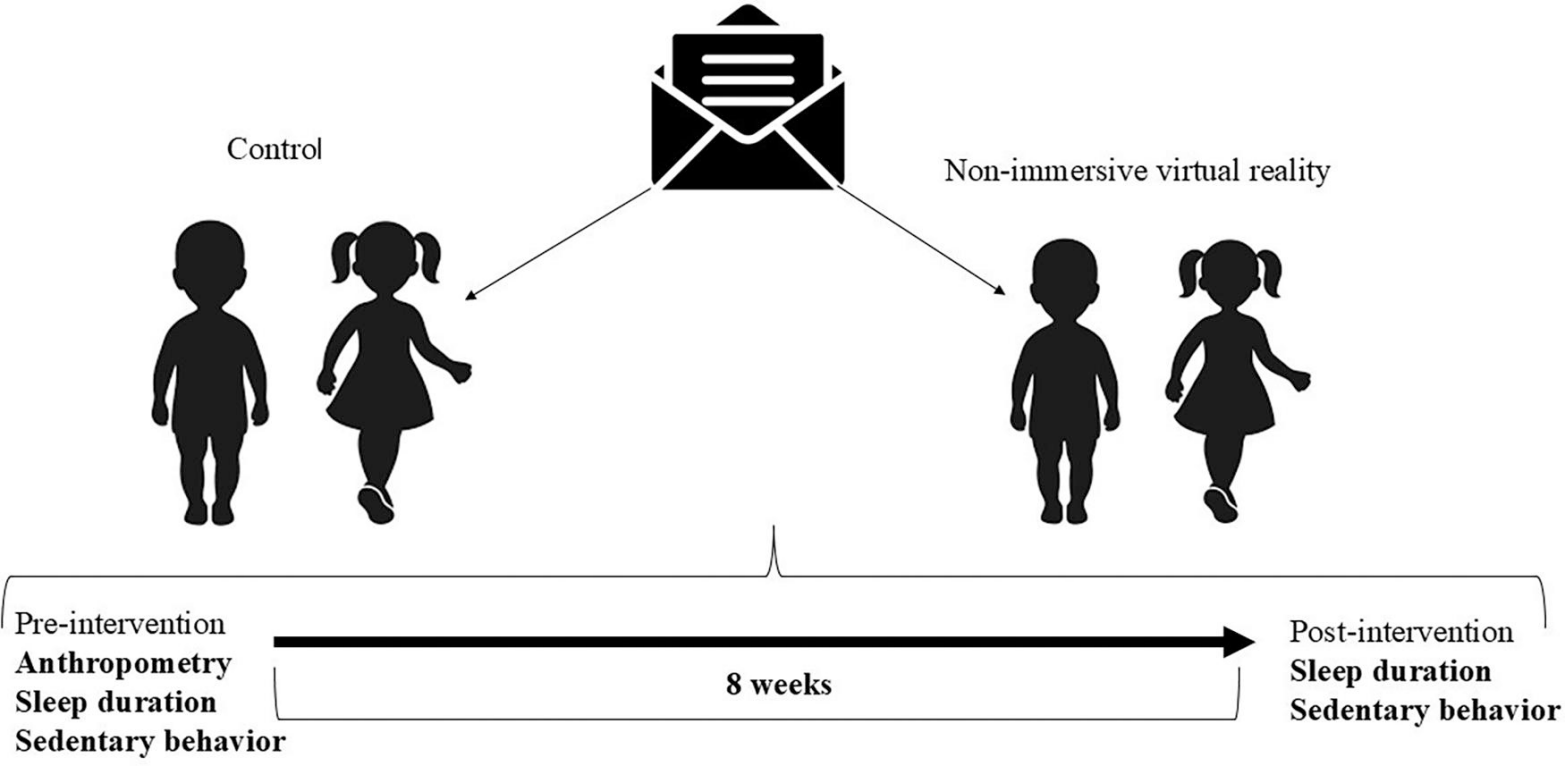
Study design and timeline of the experimental protocol.

Following randomization, baseline assessments were conducted during the week before the start of the intervention to verify initial comparability between groups. Age was subsequently controlled for in the analyses to minimize potential selection bias. The intervention period lasted 8 weeks. During this time, the experimental group participated in the proposed intervention, whereas the control group continued their usual school activities and did not receive any additional intervention. All outcome variables were assessed at two time points: (1) pre-intervention (baseline), conducted during the week preceding the intervention, and (2) post-intervention, performed during the week immediately following the completion of the intervention period. The same assessment procedures were applied to both groups at both time points. Figure 1 illustrates the study design, including the randomization process, intervention period, and assessment time points.

### 2.2 Data collection instruments anthropometry

Body weight was measured using a digital anthropometric scale (Welmy W200A LED, São Paulo, Brazil), with children wearing light clothing and no shoes. Care was taken to ensure that participants carried no objects in their pockets or on their heads. Height was assessed using a stadiometer (Welmy W200A LED, São Paulo, Brazil), which has a maximum capacity of 220 cm and millimeter precision. Weight and height classifications followed the reference standards established by the World Health Organization (WHO, 2006) and the Brazilian Ministry of Health (2008). Nutritional status was assessed using the WHO AnthroPlus software, version 2.0 (2007).

**Sleep duration**: Sleep duration was assessed using a four-item questionnaire validated for South American children aged 3 to 10 years (15). This instrument was adapted from the Children's Chronotype Questionnaire and is easy to administer and cost-effective for assessing sleep duration and sleep habits in school-aged children. Parents answered two questions: “During weekdays, how many hours (and minutes) does your child usually sleep?” and “During weekends, how many hours (and minutes) does your child usually sleep?” Sleep duration was calculated as [(weekday sleep duration  ×   5) + (weekend sleep duration  ×   2)]/7.

**Sedentary behavior time**: Sedentary behavior time was assessed using the South American Youth Cardiovascular and Environmental (SAYCARE) questionnaire, which demonstrates acceptable applicability and reliability for children and adolescents aged 3 to 18 years in South America (16). Parents are asked to fill out parts of the questionnaire that ask about how much time their kids spend doing sedentary activities like passive play (like painting, playing with dolls, or using the computer). Sedentary behavior time was calculated as [(sedentary behavior on weekdays  ×   5) + (sedentary behavior on weekends  ×   2)]/7.

### 2.3 Application of the non-immersive virtual reality protocol

All study procedures were conducted on school premises outside regular class hours. Before data collection, participants attended an introductory session in which trained research assistants demonstrated the use of the virtual reality equipment. Children were also allowed to familiarize themselves with each game. Participants were instructed to avoid vigorous physical activity on the day before and the day of each assessment or intervention session.

VR technologies are commonly classified as immersive or non-immersive. Immersive VR involves head-mounted displays equipped with sensors that capture users' orientation and movements, enabling three-dimensional interaction and a high level of perceived presence and sensory immersion. In contrast, non-immersive VR relies on conventional screens and motion-sensing devices, allowing interaction with virtual environments while maintaining simultaneous engagement with the physical world. In this study, the Xbox 360 Kinect® was used as a non-immersive, motion-based interactive system. Each session lasted 40 min and was conducted twice a week, on non-consecutive days, over eight weeks. The protocol included games designed to promote movements such as lateral jumps, lateral and anteroposterior displacements, upper limb elevation, squat jumps, and variations in movement speed. Each session was structured into three phases: five minutes of warm-up and stretching, followed by 30 min of active games delivered through virtual reality. Participants' body movements were tracked and represented by an avatar projected onto a wall using an image projector. The session concluded with five minutes of relaxation. Children exercised in pairs in a recreational, non-competitive environment, with one instructor supervising each session. For safety monitoring, blood pressure, oxygen saturation, and heart rate were measured before and after each session.

The VR games used during training included *Kinect Adventures*, *Kinect Sports*, and *Just Dance*. Progression was achieved by increasing game difficulty levels, which required greater movement complexity. Participants who missed three consecutive sessions were excluded from the intervention. After completion of the protocol and outcome assessments, children in the control group were invited to participate in the VR activities.

### 2.4 Sample size calculation

Sample size estimation was performed using G*Power software (version 3.1.9.4) for a repeated-measures ANOVA, assuming an alpha level of 0.05, statistical power of 0.80, and a medium effect size (f = 0.20). The analysis indicated that a minimum of 52 participants was required across the two groups. To strengthen the analysis of longitudinal and repeated-measures data, a linear mixed-effects model was subsequently applied, which is consistent with and supports the original sample size calculation.

### 2.5 Statistical analysis

Statistical analyses were conducted using Jamovi software (version 2.7.6). Fisher's exact test was used to compare categorical variables between groups. For sleep duration and sedentary behavior time, linear mixed-effects models were applied to evaluate differences between groups over time, including time (pre and post), group (virtual reality and control), and the time-by-group interaction as fixed effects (17). Age was included as a covariate to control for potential confounding effects. Subgroup analyses by sex were not performed due to the limited sample size, which would not provide reliable estimates, and because no prior hypotheses suggested sex-specific effects. Model assumptions, including normality of residuals, homogeneity, and independence of errors, were examined and met. Statistical significance was set at *p* < 0.05.

## 3 Results

### 3.1 Sample profile

The study was conducted in five schools, with 80 children initially assessed for eligibility. Ten children withdrew from the study due to their parents' lack of time, resulting in a final enrollment of 70 participants, who were allocated to either the VR group or the control group. During the follow-up period, six children from the VR group were excluded due to repeated absences, and two children from the control group did not attend the final assessment. Consequently, the final sample comprised 62 children, with a mean age of 6.74 ± 1.51 years.

Regarding age distribution, 19 children were five years old, 11 were six years old, 11 were seven years old, 9 were eight years old, and 12 were nine years old. Of the total sample, 29 participants were female, representing 46.8% of the sample. The mean body mass index (BMI) was 16.3 ± 3.19 kg/m^2^ in the control group and 16.4 ± 3.17 kg/m^2^ in the VR group. Socioeconomic characteristics were comparable between groups, as confirmed by Fisher's exact test (Table 1). No adverse events were reported by the children during the assessment procedures or throughout the VR sessions.

**Table 1:** Socioeconomic characteristics of the sample.

VARIABLES	*n*(%)		*p*-value (Fisher’s exact test)
Sex	Control (*n*)	Virtual Reality(*n*)	
Male	16 (25.8)	17 (27.4)	0.617
Female	12 (19.4)	17 (27.4)	
Total	**28**	**34**	
Ethnicity (self-declared)			
White	4 (7.4)	8 (14.8)	0.418
Brown	21 (38.9)	20 (37)	
Black	0	1 (1.9)	
Did not declare	3	5	
	**28**	**34**	
Number of people living in the house			
1 to 3 people	8 (13.8)	15 (25.9)	0.283
4 to 7 people	18 (31)	17 (29.3)	
Not informed	2	2	
	**28**	**34**	
Housing			
Owned	9 (15.5)	12 (20.7)	0.550
Rented	13 (22.4)	18 (31)	
Ceded	4 (6.9)	2 (3.4)	
Not informed	2	2	
	**28**	**34**	
Mother’s level of education			
Elementary school	17 (30.9)	11 (20)	0.105
Completed high school	7 (12.7)	16 (29.1)	
Completed higher education	2 (3.6)	2 (3.6)	
Not informed	2	5	
Total	**28**	**34**	
Family income			0.068
No income	14 (23.7)	8 (13.6)	
Up to 1 minimum wage	9 (15.3)	19 (32.2)	
From 1 to 3 minimum wages	3 (5.1)	6 (10.2)	
Not informed	2	1	
	**28**	**34**	

### 3.2 Effects of the intervention

The VR intervention did not show a significant main effect on the evaluated variables (Table 2). The observed effect sizes (*η*^2^p) were primarily small for both outcomes assessed. Therefore, even in cases where results approached statistical significance, the magnitude of the effects suggests that the intervention had a limited impact. Additionally, standardized effect sizes derived from the model indicated negligible interaction effects (Cohen's d ≈ 0.00), reinforcing the minimal magnitude of the intervention effects.

**Table 2:** Analysis of the main effects and interaction effects in the linear mixed model.

Sleep duration	F	df	*p*	*η^2^p*
Time	0.776	1	0.380	0.006
Group	1.883	1	0.173	0.016
Age	3.198	1	0.076	0.026
Time ✻ Group	0.041	1	0.839	0.000
Sedentary behavior time				
Time	2.972	1	0.088	0.027
Group	3.347	1	0.070	0.030
Age	0.119	1	0.731	0.001
Time✻ Group	0.018	1	0.895	0.000

*η^2^p* = partial eta squared. Effect size interpretation: small (0.01), medium (0.06), and large (0.14).

The analysis of the model estimates indicated that the VR group exhibited, on average, 19 min less sleep duration than the control group (SE = 14.34; 95% CI: −48.08 to 8.72); however, this difference was not statistically significant (*p* = 0.173) (Table 3). With respect to sedentary behavior time, the VR group presented an average of 49 additional minutes compared with the control group, although this difference also did not reach statistical significance (*p* = 0.070).

**Table 3:** Estimates of the parameters of the mixed linear model (sleep duration and sedentary behavior time).

			95% Confidence Intervals			
Effect	Estimate	SE	lower	Upper	t	*p*
Sleep duration						
VR - C	−19.680	14.341	−48.077	8.716	−1.372	0.173
Age	−8.497	4.752	−17.905	0.912	−1.788	0.076
post - pre	−12.258	13.914	−39.809	15.292	−0.881	0.380
(VR - Control) ✻ (post - pre)	−5.660	27.828	−60.761	49.442	−0.203	0.839
Sedentary behavior time						
VR - C	49.415	27.010	−4.119	102.949	1.829	0.070
Age	−3.048	8.852	−20.593	14.497	−0.344	0.731
post - pre	−45.188	26.212	−97.140	6.764	−1.724	0.088
(VR - Control) ✻ (post - pre)	6.936	52.425	−96.968	110.840	0.132	0.895

The estimated marginal means (in minutes), derived from the age-adjusted mixed-effects model, for each group illustrate changes over time (Table 4). Mean sleep duration in both groups was within the recommended range for this age group, at approximately 10 h per day. In contrast, sedentary behavior time was high in both groups, with estimated marginal mean values of 340 min in the control group and 390 min in the VR group. A descriptive reduction in sedentary behavior time from pre- to post-intervention was observed, consistent with the marginal *p*-value for the time effect (*p* = 0.088).

**Table 4:** Estimated averages according to group (C and VR) and time (pre- and post-intervention).

Sleep duration (minutes)			95% Confidence Intervals	
Group/Time	Mean	SE	Lower	Upper
Control	620 (10 h 20 min)	10	600	641
VR	601 (10 h 01 min)	9	582	620
pre	617 (10 h 17 min)	9	597	636
post	605 (10 h 05 min)	9	585	624
Sedentary behavior time (minutes)			95% Confidence Intervals	
Control	340 (5 h 40 min)	19	301	380
VR	390 (6 h 30 min)	17	355	425
pre	388 (6 h 28 min	18	351	424
post	342 (5 h 42 min)	18	306	379

## 4 Discussion

The present study aimed to evaluate the effects of a non-immersive virtual reality game–based protocol on sleep duration and sedentary behavior in children. The findings indicated that the intervention did not produce statistically significant changes in the outcomes assessed over time. Both groups reported average sleep durations within recommended ranges; however, high levels of sedentary behavior were observed in both the control and intervention groups.

The groups were comparable in terms of socioeconomic characteristics. The sample consisted predominantly of mixed-race children, as reported by their parents, living in rented housing, with household sizes ranging from three to seven members. Most mothers had completed elementary education, and the reported family income was up to one Brazilian minimum wage (R$1,518.00). Previous research has shown that healthier sleep patterns— characterized by longer duration, better quality, and earlier sleep onset—during childhood and adolescence are associated with improved cognitive and academic outcomes. Importantly, the strength of these associations may vary according to socioeconomic status and race/ethnicity (18).

Sleep habits change across the lifespan. In early childhood, sleep patterns are typically aligned with a morning-oriented circadian profile (19). During middle childhood, these patterns tend to remain relatively stable but are influenced by school schedules, extracurricular demands, and parental bedtime routines (20). As children enter puberty, a shift toward a later circadian phase is commonly observed (19). In the present study, children demonstrated average sleep durations within age-appropriate recommendations. However, future studies including a wider age range and greater variability in sleep duration may be better positioned to detect potential effects of virtual reality–based interventions.

The non-immersive virtual reality intervention did not result in statistically significant changes in sleep duration or sedentary behavior time. Nevertheless, these findings remain informative, as they suggest that the intensity and duration of the protocol may not have been sufficient to elicit measurable effects. Consistent with this, evidence in adults indicates that habitual (up to 7 h per week) or occasional use of electronic games may have positive effects on sleep when appropriately implemented, and cognitively demanding games may improve sleep quality in terms of continuity, stability, and organization (21). These results support the notion that regular and moderate use of video games may not negatively affect sleep, complementing our findings in children. Previous research indicates that longer school-based interventions can positively influence sleep-related outcomes. For example, a study involving 120 children aged 8 to 11 years implemented a gamification-based protocol over 12 weeks, while the control group continued with traditional physical education classes (22). The intervention group demonstrated reductions in anxiety and improvements in sleep quality, as assessed by the Children's Sleep Habits Questionnaire. The authors highlighted the potential of game-based strategies as effective interventions within primary school settings. Similarly, extended reality games have been reported to be easy to use, well accepted by typically developing children, and capable of eliciting positive emotional responses (23).

Regarding physical activity, a study encompassing twenty systematic reviews evaluated the evidence on the effectiveness of active video games in promoting physical activity across diverse populations (24). Collectively, the included studies comprised more than 180,000 participants. The Xbox Kinect was identified as the second most frequently investigated platform, with Kinect Sports and Kinect Adventures ranking among the most commonly utilized games. Across these investigations, virtual reality–based games consistently elicited light to moderate intensity levels during gameplay, corresponding to approximately 3–6 metabolic equivalents (METs). Another study compared the energy expenditure and intensity of active video games with treadmill walking in children and adolescents of both sexes (aged 8–13 years) enrolled in public schools. Energy expenditure and heart rate were measured at rest, during walking at 3 km/h, 4 km/h, and 5 km/h, and while playing active games (Adventure, Boxing I, Boxing II, and Dance). Physical activity levels were assessed using an accelerometer (25). The results indicated that Xbox 360 Kinect games elicited energy expenditure and moderate-intensity physical activity in both sexes. The authors highlighted active video games as a promising strategy for increasing physical activity levels. In the present study, the intensity of the games included in the protocol was not assessed; heart rate was monitored solely for supervision purposes during the intervention. This measure could have been useful to better understand the relationship between activity intensity and the absence of an effect on sleep duration, especially since previous studies demonstrating physiological adaptations frequently employ interventions with higher weekly frequencies (≥3 sessions per week) (22, 24, 25), which may provide a greater stimulus for sleep-related outcomes. Physical activity levels, like sleep, are essential for children's health and development. In the present study, both groups exhibited high average sedentary behavior time. This finding is concerning, as engaging in sedentary activities for more than 120 min per day has been associated with an increased risk of overweight and obesity in childhood compared with lower levels of sedentary behavior (26). Supporting this concern, a qualitative study based on 24 semi-structured interviews with caregivers of children under five years of age living in poverty in Mexico examined physical activity practices and sedentary behavior (27). The results revealed limited promotion of physical activity by caregivers, gender-related differences influenced by cultural factors, and a lack of accessible, free, and safe spaces for physical activity. Caregivers also associated sedentary behavior with children's individual characteristics and the frequent use of mobile phones and tablets.

Virtual reality has been increasingly recognized as a promising tool for promoting physical activity among children and adolescents (28). In this population, daily routines are strongly shaped by family and school environments. For instance, one study examined the effects of a physical activity intervention supported by a non-immersive VR gaming system on sedentary behavior, physical activity levels, and cognitive skills in 13 preschool children (29). During school recess, participants engaged in VR-based games for 30 min, one to two times per week, over six weeks (eight sessions in total). Physical activity levels were objectively assessed using accelerometers.

Research has shown that implementing this type of strategy during school recess can help preschool children achieve the recommended daily target of 60 min of physical activity. In addition, engaging in VR-based physical activity for as little as 30 min per day has been associated with improvements in cognitive functioning. The present study included a broader age range, a larger sample size, and a longer intervention period; however, outcome assessment relied on subjective measures obtained through questionnaires. Simple modifications to daily routines may help prevent future health problems. To improve health-related quality of life, it is essential to reduce screen time and increase sleep duration (30). Early investigation of children's habits is crucial for implementing interventions that promote positive health behaviors, which may also persist into adulthood.

This study has some limitations. The intervention duration of eight weeks may have been insufficient to produce statistically significant effects. Given the high average sedentary behavior time observed among participants, it is possible that a greater number of sessions or a larger sample size would be necessary to detect meaningful changes. The intervention totaled 80 min per week, which is below the World Health Organization's recommendation of 60 min of moderate-to-vigorous physical activity per day. Moreover, the intensity of the activities performed was not objectively measured. As a result, it is not possible to determine whether participants reached a sufficient physiological stimulus to induce changes in sleep outcomes, which may partly explain the absence of significant effects observed in this study. Due to limited resources, objective assessment methods such as actigraphy or accelerometry could not be employed. As a result, the study is subject to potential recall bias, which is inherent to self-report instruments. To mitigate this limitation, short recall periods and objective, structured questions were used, and all questionnaires applied were validated for the target population. In the present study, additional sleep parameters, such as sleep latency and sleep efficiency, were not evaluated, although they may represent more sensitive indicators of intervention-related changes. Furthermore, baseline sleep duration was already within the recommended range for age, which may have produced a ceiling effect, thereby reducing the ability to detect additional improvements. The observed effect sizes were small, suggesting that future studies should consider more intensive intervention protocols and, when feasible, incorporate objective monitoring of sleep and physical activity variables. Importantly, the absence of statistically significant effects helps reduce publication bias and enhances understanding of the parameters influencing this type of intervention.

Overall, the findings in this sample indicate that the non-immersive virtual reality game-based protocol did not affect sleep duration or sedentary behavior time in children. The lack of observed changes may be related to the intervention dose and to the sensitivity of the self-report instruments used. Participants exhibited high levels of sedentary behavior, underscoring the need for intervention strategies to improve sleep habits, reduce sedentary time, and promote physical activity, particularly within school settings.

Importantly, the VR-based protocol proved to be feasible, easily implementable in the school environment, and demonstrated good adherence among both children and caregivers. These findings represent important preliminary evidence supporting the feasibility and acceptability of virtual reality-based interventions in school contexts. Virtual reality games may represent a viable and well-accepted approach for increasing children's interest and engagement in physical activity within educational contexts. Future research should investigate longer and multi-component interventions, combining VR-based activities with family- or curriculum-based physical activity strategies and incorporating objective measures such as accelerometry or actigraphy to better capture behavioral changes and their potential impact on children's movement behaviors.

## Data Availability

The original contributions presented in the study are included in the article/supplementary material, further inquiries can be directed to the corresponding author.
